# Gender-Specific Combination HIV Prevention for Youth in High-Burden Settings: The MP3 Youth Observational Pilot Study Protocol

**DOI:** 10.2196/resprot.5833

**Published:** 2017-03-08

**Authors:** Jasmine Buttolph, Irene Inwani, Kawango Agot, Charles M Cleland, Peter Cherutich, James N Kiarie, Alfred Osoti, Connie L Celum, Jared M Baeten, Ruth Nduati, John Kinuthia, Timothy B Hallett, Ramzi Alsallaq, Ann E Kurth

**Affiliations:** ^1^ New York University College of Nursing Global New York, NY United States; ^2^ University of Nairobi Kenyatta National Hospital Nairobi Kenya; ^3^ Impact Research & Development Organization (IRDO) Kisumu Kenya; ^4^ Kenya Ministry of Health National AIDS & STI Control Program (NASCOP) Nairobi Kenya; ^5^ Department of Obstetrics and Gynecology Kenyatta National Hospital University of Nairobi Nairobi Kenya; ^6^ University of Washington School of Public Health Seattle, WA United States; ^7^ Imperial College London Department of Infectious Disease Epidemiology London United Kingdom; ^8^ New York University College of Nursing Global, New York, NY and Yale School of Nursing New Haven, CT United States

**Keywords:** HIV, combination prevention, Kenya, youth, PrEP, VMMC, mobile health, family planning, cash transfer, biometrics

## Abstract

**Background:**

Nearly three decades into the epidemic, sub-Saharan Africa (SSA) remains the region most heavily affected by human immunodeficiency virus (HIV), with nearly 70% of the 34 million people living with HIV globally residing in the region. In SSA, female and male youth (15 to 24 years) are at a disproportionately high risk of HIV infection compared to adults. As such, there is a need to target HIV prevention strategies to youth and to tailor them to a gender-specific context. This protocol describes the process for the multi-staged approach in the design of the MP3 Youth pilot study, a gender-specific, combination, HIV prevention intervention for youth in Kenya.

**Objective:**

The objective of this multi-method protocol is to outline a rigorous and replicable methodology for a gender-specific combination HIV prevention pilot study for youth in high-burden settings, illustrating the triangulated methods undertaken to ensure that age, sex, and context are integral in the design of the intervention.

**Methods:**

The mixed-methods, cross-sectional, longitudinal cohort pilot study protocol was developed by first conducting a systematic review of the literature, which shaped focus group discussions around prevention package and delivery options, and that also informed age- and sex- stratified mathematical modeling. The review, qualitative data, and mathematical modeling created a triangulated evidence base of interventions to be included in the pilot study protocol. To design the pilot study protocol, we convened an expert panel to select HIV prevention interventions effective for youth in SSA, which will be offered in a mobile health setting. The goal of the pilot study implementation and evaluation is to apply lessons learned to more effective HIV prevention evidence and programming.

**Results:**

The combination HIV prevention package in this protocol includes (1) offering HIV testing and counseling for all youth; (2) voluntary medical circumcision and condoms for males; (3) pre-exposure prophylaxis (PrEP), conditional cash transfer (CCT), and contraceptives for females; and (4) referrals for HIV care among those identified as HIV-positive. The combination package platform selected is mobile health teams in an integrated services delivery model. A cross-sectional analysis will be conducted to determine the uptake of the interventions. To determine long-term impact, the protocol outlines enrolling selected participants in mutually exclusive longitudinal cohorts (HIV-positive, PrEP, CCT, and HIV-negative) followed by using mobile phone text messages (short message service, SMS) and in-person surveys to prospectively assess prevention method uptake, adherence, and risk compensation behaviors. Cross-sectional and sub-cohort analyses will be conducted to determine intervention packages uptake.

**Conclusions:**

The literature review, focus groups, and modeling indicate that offering age- and gender- specific combination HIV prevention interventions that include biomedical, behavioral, and structural interventions can have an impact on HIV risk reduction. Implementing this protocol will show the feasibility of delivering these services at scale. The MP3 Youth study is one of the few combination HIV prevention intervention protocols incorporating youth- and gender-specific interventions in one delivery setting. Lessons learned from the design of the protocol can be incorporated into the national guidance for combination HIV prevention for youth in Kenya and other high-burden SSA settings.

**Trial Registration:**

ClinicalTrials.gov NCT01571128; http://clinicaltrials.gov/ct2/show/NCT01571128?term=MP3+youth&rank=1 (Archived by WebCite at http://www.webcitation.org/6nmioPd54)

## Introduction

Nearly three decades into the epidemic, sub-Saharan Africa (SSA) remains the region most heavily affected by human immunodeficiency virus (HIV), with nearly 70% of the 34 million people living with HIV globally residing in the region [1-4]. Youth aged 15 to 24 years bear the highest burden of new infections, and in SSA, account for 80% of the 1.9 million new infections each year [5,6]. Young females are twice as likely as their male counterparts to be infected [4,6], making females 15 to 24 years in SSA the most at-risk group for HIV infection. The incidence and prevalence of HIV among youth in SSA remains high [4] and literature shows that programs for youth are often vertical, uncoordinated, and not evidence-based [7,8]. The delivery of proven interventions needs to account for the complexity of the interconnected drivers of HIV, especially among youth, necessitating combination prevention packages relevant to the target population(s). We set out to develop a protocol for a cross-sectional and longitudinal pilot study of a sex- and gender-specific combination HIV prevention approach in western Kenya for youth called “MP3 Youth.” The focus of this paper is to outline the development details of the protocol, which includes a multi-method approach for designing a pilot study of combination HIV prevention.

### HIV in Kenyan Youth

**Figure 1:**
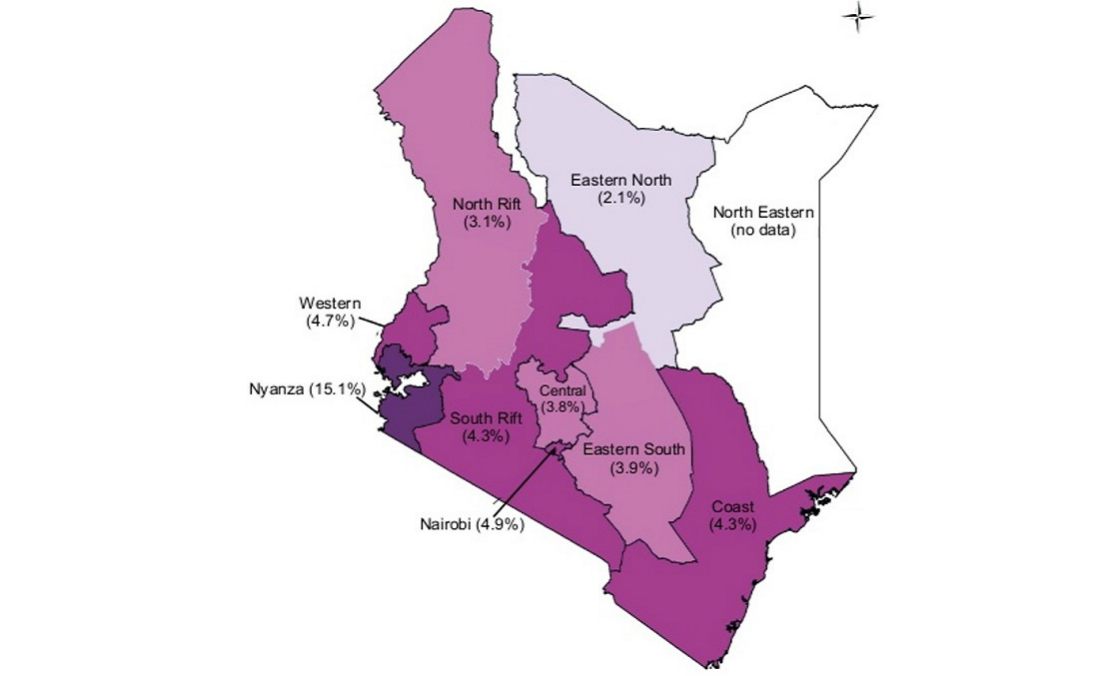
Map of HIV prevalence in Kenya (KAIS, 2014).

The study setting for this protocol is Kenya, where national HIV prevalence is 5.6% among individuals aged 15 to 49 years [9,10]. With the exception of the Nyanza Province where the study will take place, HIV prevalence in Kenya has declined [9-11]. The prevalence of HIV varies markedly by region, and ranges between 2% to 15% (Figure 1) [11,12]. Children and youth aged 0 to 24 years represent 55.6% of Kenya’s population [12] and surveillance data indicates that most new HIV infections occur among youth 15 to 24 years of age [11,12]. Females between the ages of 20 to 24 have a risk of HIV infection, four times higher than males of the same age [10-12]. The 2012 Kenya AIDS indicator Survey (KAIS) shows that nationally the prevalence of HIV among females aged 15 to 19 is 1.1%, a reduction from the 3.5% reported in 2007. The HIV prevalence for females aged 20 to 24 years is 4.6% (previously 7.5%) [12]. The prevalence of HIV among males is much lower than females of the same age group. HIV prevalence among males aged 15 to 19 and 20 to 24 years old is 0.9% and 1.3%, respectively [10,11].

### Theoretical Basis for Combination HIV Prevention Package

**Figure 2:**
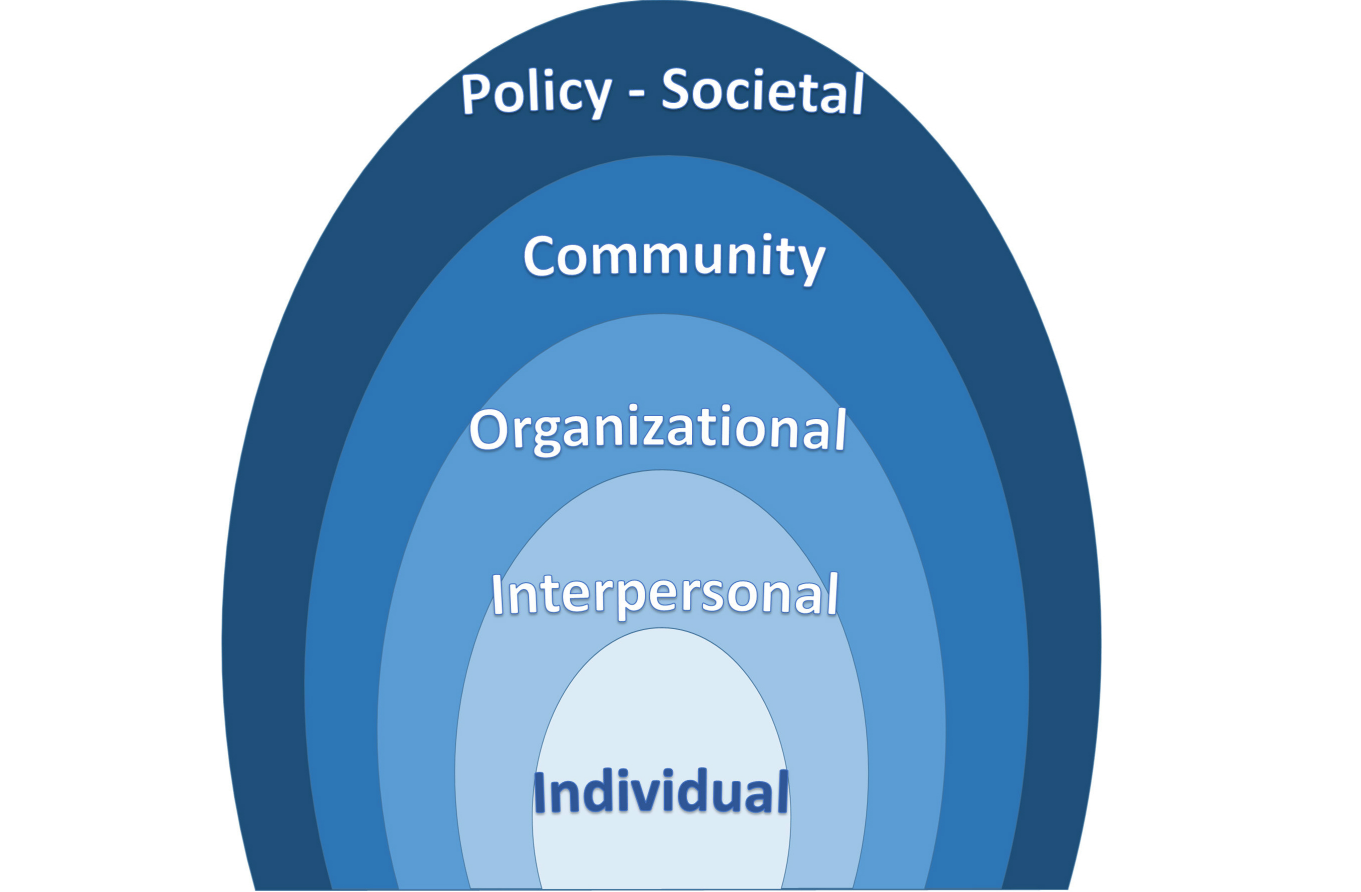
Social ecological framework (Centers for Disease Control and Prevention, 2013).

The evidence-based methodology for the MP3 Youth combination intervention protocol is guided by a multi-theoretical approach grounded in social ecological theory (Figure 2) [13,14]. The combination prevention approach needs to address behavioral, biomedical, and structural levels, and tailor interventions to address each level according to age and sex. MP3 Youth focuses on the context in which an individual finds him or herself socially and physiologically. The prevention framework includes reducing health risks associated with age, gender, and biological susceptibility. HIV prevention requires not only equipping youth with knowledge, skills, risk perception, condoms, and positive social norms, but also instituting systems change [15]. Systems change includes policies that prioritize youth sexual and reproductive health across sectors. MP3 Youth builds upon these psychosocial determinants of HIV prevention and consolidates the prominent themes into the MP3 Youth framework. The social ecological theory guides MP3 Youth to address the gender-specific risks across this broad spectrum of factors from individual to structural (including economic factors) [13,14]. The final package of interventions to be selected for inclusion in the protocol meets all aspects of the social ecological framework.

The interventions will be offered in a mobile health setting. Mobile health events involve determining an appropriate place in the community and setting up a temporary health center using vans, tents, or other temporary structures. Mobile events are effective for reaching rural and other hard-to-reach populations. The mobile health setting will take into account the socio-political and community context of the participants. This context will drive the location, time, and manner in which the interventions are offered.

The four aims of the MP3 Youth study are shown in Textbox 1. Each of the aims has specific analytic objectives and outcomes. This paper will focus on the development of the objectives and outcomes for Aim 3 (the pilot study). We hypothesize that by strategically designing and piloting a gender- and youth-specific combination HIV prevention intervention, we can learn important lessons about scaling up combination prevention to curb the HIV epidemic among young people.

**Aims of the MP3 Youth study.**

Aim

- Aim 1: Select a package of interventions by identifying epidemiologic targets for HIV prevention among youth in sub-Saharan Africa through a systematic review, meta-analysis, and qualitative data collection.
- Aim 2: Perform mathematical modeling for selection of interventions with the highest population impact.
- Aim 3: Develop a protocol and implement a pilot study of a gender-specific combination HIV prevention package for youth (aged 15 to 24) in western Kenya.
- Aim 4: Derive lessons from the pilot for future combination HIV prevention evaluations.

## Methods

**Figure 3:**
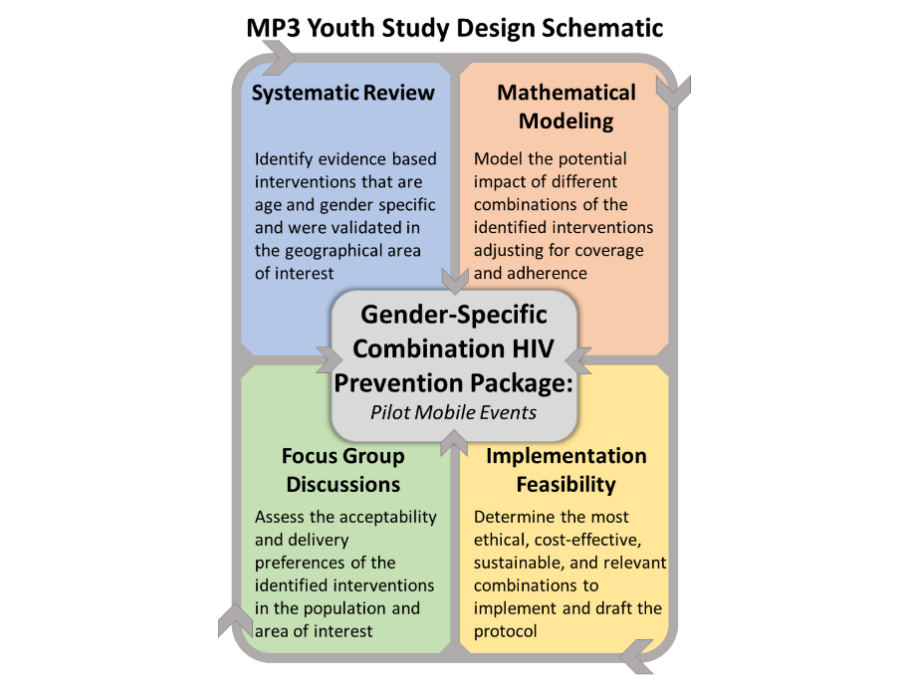
MP3 Youth Study Design Schematic.

MP3 youth is a mixed-methods study incorporating quantitative, qualitative, and modeling approaches, and capturing cross sectional and longitudinal data elements. A multi-method process was undertaken to design the MP3 Youth combination HIV prevention pilot study protocol (Aim 3) (Figure 3). To determine the most effective evidence-based interventions for youth HIV prevention in SSA, we first conducted a systematic review of the literature. In addition to the evidence, the study collects formative data through focus groups to determine the knowledge, attitudes, and preferences of youth about which interventions are most acceptable, as well as by whom and where they would like them to be delivered. Mathematical modeling will be used to determine the impact of the interventions, considering coverage and adherence levels, as well as to determine the most cost-effective combination of interventions (Figure 3). All of these methods are useful for determining the best “package” of gender-specific interventions during the intervention design phase. The steps for developing the multi-method protocol for the field pilot study (Aim 3) are outlined below.

### Aim 1: Review and Selection of Package Components Procedures

**Figure 4:**
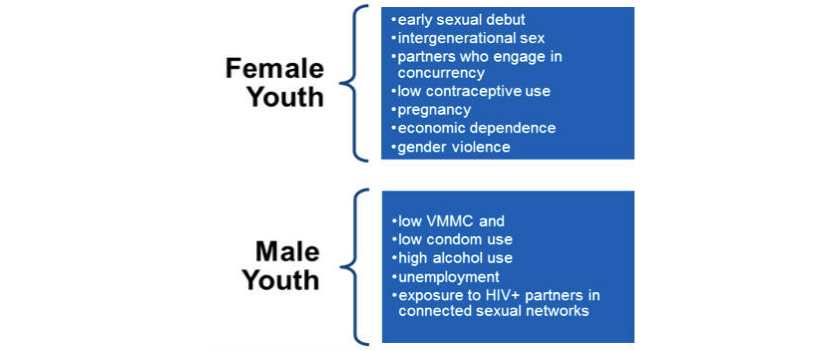
Key drivers of youth HIV risk in sub-Saharan Africa.

To develop the protocol, our team compiled a priori knowledge of what drivers may be most important to target for HIV prevention among SSA youth (Figure 4). These include early initiation of sex, lack of circumcision, alcohol abuse, power structure (intergenerational sex, gender violence), lack of HIV serostatus knowledge, low contraceptive use, and high rates of unintended pregnancy. Our approach was to understand not just modifiable drivers of HIV transmission among youth, but also to lay out a replicable strategy for assessing whether there were sufficient evidence-based interventions to address each driver. We constructed a framework with “strength of evidence” thresholds to determine whether to include specific interventions in the combination package for the protocol.

#### Systematic Review

The development of the combination package included a systematic review and meta-analysis where we conducted a systematic review using standardized procedures [16]. The review question (gender-specific modifiable drivers of the epidemic and evidence-based interventions among youth) yielded a critical base of evidence that fed into the development of the focus group discussions, the mathematical modeling, and the overall protocol design. While a detailed account of the methods and results of the systematic review will be reported in a separate publication, they were mentioned here to show the groundwork for the pilot study protocol elements.

#### Focus Group Discussions

The development of this protocol includes an appreciation of the sociocultural and community context in which youth experience. Community context is critical to a comprehensive protocol that will lead to successful pilot study implementation. To assess the acceptability of the components in the pilot study protocol, we collected qualitative data using focus groups with youth and community leaders to assess HIV risk and protective factors and the best location and format for the delivery of the combination prevention interventions. Topic guides for these groups were informed by the systematic review and key stakeholders. The detailed methods and results of the focus group discussion will be outlined in a separate publication. The description of this aim here is intended to illustrate the foundation upon which the pilot study protocol was built.

### Aim 2: Mathematical Modeling for Component Selection

Components from the systematic review and focus group discussions were fed into the development of a mathematical model that was used to triangulate the appropriateness and potential effectiveness of the interventions to be included in the pilot study protocol. Results of the systematic review and epidemiologic analyses were used to provide the modeling parameters. Mathematical modeling was used to assist in selecting components of intervention packages most likely to have complementary or synergistic effects. The modeling provided estimated impact and costs of strategies that focus interventions on different sub-populations according to sex, age, risk behavior, and other factors. An appropriate model structure was agreed upon following detailed consultations with the study team and reviews of the modeling literature. The model incorporated sex- and age- specific coverage to long-term interventions and risk compensation that could undermine intervention package efficacy over time. The model was designed, constructed, solved, and analyzed using modern computational software (MathWorks, MATLAB) using techniques developed by Hallett et al [17]. A user-friendly toolkit was built using MATLAB and several stakeholders were trained on how to use the modeling tool for intervention selection and program planning.

The model will be continuously updated with information generated during the implementation of the pilot study to produce more refined and robust projections of both impact and cost-effectiveness. Model projections were fed into study design considerations in Aim 3 (pilot study) and Aim 4 (the effectiveness trial). The full details of the methods and results of the modeling will be reported in another paper. The inclusion of the modeling here is intended to show the process for finalizing the component selection of a combination HIV prevention package described in depth in this protocol.

### Aim 3: Pilot Study

The previous aims were completed to inform Aim 3 of this protocol. This section provides an overview of setting, participants, and details of how interventions can be implemented and how data should be collected. Our preliminary research confirmed that there were numerous examples of individual HIV prevention interventions aimed at addressing the drivers of the HIV epidemic among youth [18-30]. Many of the vertically-focused single interventions were efficacious, but there remained a need for combination approaches [8]. The studies reviewed as part of the systematic review informed our decision about the most effective and feasible components to include in the protocol for the combination package. These components are HIV testing and counseling (HTC) [31,32], condoms [33,34], family planning (contraception) [35,36], voluntary medical male circumcision [37-39], conditional cash transfers (CCTs) [40-44], pre-exposure prophylaxis (PrEP) [40,45,46], and HIV care and treatment [47,48]. All interventions included in the protocol to be offered as part of MP3 Youth were chosen because they were efficacious, youth-friendly, possible to implement in a mobile health setting, and potentially sustainable following the MP3 Youth study.

#### MP3 Youth Package

**Figure 5:**
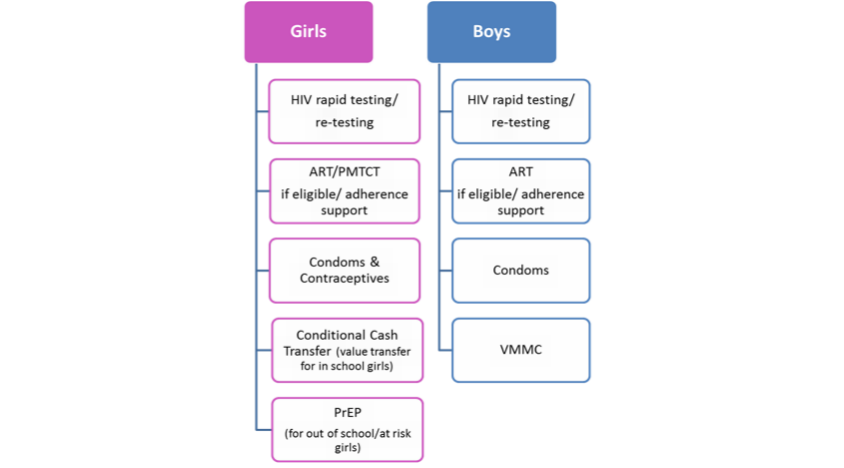
MP3 Youth packages.

Based on results of the first two aims, the gender-specific MP3 Youth package was developed (Figure 5). The tailoring of interventions to specific individuals is referred to as the “MP3 Youth package of services.” For all participating youth, the package will include HIV counseling and testing. For all HIV-positive participants, the package will include point of care cluster of differentiation 4 (CD4) and viral load testing, and facilitated linkage to care (n is approximately 100), including prevention of mother-to-child transmission for pregnant females. For all participating males, the package will also include condoms and voluntary medical male circumcision, whereas for all participating females the package will also include contraception (both male and female condoms) and family planning. PrEP will be included for HIV-negative, out-of-school females while CCT will be included for HIV-negative, in-school females. The protocol aims for approximately 1000 youth to be enrolled in the pilot study and from these, sub-cohorts of youth (approximately 300) will be followed up prospectively for 12 months to document behaviors and adherence related to selected interventions. In addition, follow-up by phone to approximately 100 youth who are HIV negative at baseline will assess whether or not they were HIV retested over the last 12 months or if they are willing to be retested at the 12-month follow-up period.

The combination MP3 Youth package menu options will be delivered through community-based mobile health teams and all interventions will be offered on-site. PrEP and CCT cohort enrollment will also be initiated on site and followed-up prospectively off-site. Mobile health teams will be able to select the most appropriate location within a community to set up tents to deliver the MP3 Youth packages. Mobile health events increase accessibility to the interventions and reduces stigma by offering it in the context of a health event where other youth-friendly services are also being provided [32,49].

#### Study Objectives

The objectives of the pilot study were developed based on the formative stages of the protocol development by an expert panel that recognized the gaps that this study could fill. The primary objective is to determine the feasibility and acceptability of a pilot combination HIV prevention package specific for female and male youth (MP3 Youth) in Homa Bay County, Nyanza Province, Kenya. This will be determined by examining uptake and coverage of the intervention package among youth. The secondary objectives are (1) to examine adherence to HIV care including antiretroviral therapy (ART) among HIV-positive males and females 15 to 24 years of age; (2) to evaluate the feasibility of offering PrEP to high HIV risk out-of-school females 18 to 24 years of age and to contribute data to the current question of how best to initiate and support ongoing adherence for females using PrEP; (3) to assess the feasibility of delivering CCT to keep girls in school over the 12 months; and (4) to evaluate school attendance over 12 months of CCT cohort participants. For the logic model, see Multimedia Appendix 1.

#### Setting

The mobile health events for intervention delivery are proposed to be held in Homa Bay County, Nyanza Province, Kenya using the infrastructure of our community partner Impact Research and Development Organization (IRDO).

#### Participants and Sample Size

In the cross-sectional portion of the study, we plan to enroll approximately 1000 youth (ages 15 to 24) over the course of 10 mobile events, held for 7 to 10 days each. We plan on enrolling a sub-section of the enrolled participants into longitudinal cohorts (approximately 300 youth).

The pilot study is not meant to provide a definitive test of the combination package efficacy. Nevertheless, power calculations can provide an idea of the magnitude of temporal effects on ART adherence or school attendance that could be reliably detected using conventional tests of significance. Assuming at least an 80% retention rate at the 12-month follow-up, a minimum of 40 CCT cohort participants, 40 PrEP cohort participants, and 80 HIV-positive cohort participants will contribute data for all planned assessments. A paired-samples *t* test with 80 pairs can detect a difference in means of one-third of a standard deviation (*d*=.33) with 83% power. A paired-sample *t* test with 40 pairs can detect a difference in means of *d*=.45, 80% power.

#### Unique Interventions

Many of the interventions selected to be offered as a part of the MP3 Youth protocol have been well documented in the literature and outlined in other papers [7,50,51]. This paper does not describe the rationale or process for HIV testing, linkage to care, family planning (condoms and contraception), or male circumcision—these processes are well known and documented—but discusses PrEP and CCT, two of the unique intervention components included in the MP3 Youth pilot study combination package. We outline our specific procedures for implementing these interventions as part of the innovation of the MP3 Youth protocol.

##### Pre Exposure Prophylaxis

For PrEP, Truvada or tenofovir disoproxil fumarate/emtricitabine (TDF/FTC) will be offered to out-of-school females aged 18 to 24 years as part of a demonstration project within this pilot study. Given that HIV prevalence for women in Kenya peaks earlier than men [11], we believe it is reasonable to provide PrEP only to eligible females aged 18 to 24 years. Our MP3 Youth mathematical modeling confirmed the additive role of PrEP as a prevention intervention component for women when offered during the highest window of HIV risk (from ages 18 to 24). The National AIDS and STI Control Program (NASCOP) in Kenya will offer PrEP as part of their prevention services as PrEP was officially approved for use in Kenya [52]. The US President’s Emergency Plan for AIDS Relief (PEPFAR) program in Kenya has also included PrEP as an intervention for the HIV prevention initiative called DREAMS [53]. This protocol will assess potential feasibility and implementation issues of offering PrEP to adolescent females in Kenya.

We will measure adherence to TDF/FTC for PrEP by employing pharmacological and non-pharmacological measures of adherence. Measures include self-report, electronic medication vials (eCAPs), and analysis of tenofovir diphosphate (TFV-DP) and emtricitabine triphosphate (FTC-TP) drug levels as assayed from dried blood spots (DBS). eCAPs record the time and date when each pill bottle is opened. This technology was successfully used in other studies [54,55]. DBS will provide biomarker data for participant adherence to TDF/FTC for PrEP. DBS is relatively easy to collect, store (within time constraints), and transport [56-58], making it an appropriate method for mobile health events.

##### Conditional Cash Transfers

CCTs will be offered to girls enrolled in school (aged 15 to 24 years) and their families as part of the MP3 Youth package. The girls will be required to attend school at least 80% of the time in order for her and her family to receive the CCT. This will be measured by visiting the schools to check attendance. Much of the CCT evidence base has come from government sponsored programs, like the Mexico Opportunidades program designed as an anti-poverty program with positive impacts on social, economic, and health indicators [59]. CCT programs have demonstrated multiple impacts, such as keeping girls in school and measurable health benefits. The National Government of Kenya, in collaboration with the United Nations Children's Fund (UNICEF) and the World Bank, initiated a program in 2002 called the “Kenya *Cash Transfer Program* for Orphans and *Vulnerable Children.”* The program transferred USD $14 to $28 per month (depending on the number of vulnerable children in the household) to vulnerable youth in Kenya [60]. A recent study by Handa and colleagues (2014) evaluated the Kenya cash transfer program and found a 31% reduction in sexual debut [61]. The MP3 Youth pilot study protocol will offer critical information on the feasibility of keeping girls in school using CCT as an intervention.

#### Cohort Follow-Up Schedules

Participants who attend the mobile MP3 Youth events will be enrolled into 4 cohorts which will be followed up for a period of 12 months via unstructured supplementary service data (USSD) and SMS text message (short message service, SMS) surveys and/or in-person computer-assisted personal interview (CAPI) surveys. The PrEP cohort will be followed up monthly for the first 6 months and every 3 months for the remaining 6 months in a mixture of in-person clinical visits and SMS and USSD surveys to facilitate adherence and conduct laboratory assessments (for HIV and pregnancy). The CCT cohort will be followed up every 3 months (0, 3, 6,9,12 months) for 12 months using an in-person CAPI and school registers checked for attendance. The HIV-positive cohort will also be followed up at intervals of 3 months via SMS and USSD surveys. A random subset of the HIV-negative youth who agree to be called in one year will receive a phone call to assess their HIV retesting behaviors and intentions. The HIV-positive cohort will undergo a clinical assessment at baseline and at month 12 (CD4 and viral load). The CCT cohort will undergo a clinical assessment at baseline and at month 12 (HIV testing and counseling and pregnancy testing). We will administer SMS and/or USSD and CAPI surveys to the cohorts to assess longitudinal self-reported behaviors. During all clinical/laboratory assessments, adverse events will be assessed.

#### Biomarkers and Adherence

Although efficacy of the individual components of the intervention package was already demonstrated in well-conducted randomized trials, we are electing to re-do CD4 and viral load levels (biomarker proxy for adherence) at 12 months in the HIV-positive cohort and collect biomarker data from the PrEP cohort to monitor adverse events and, in case of seroconversion, resistance. eCAPs and DBS will be used to monitor adherence to TDF/FTC. This combination of self-report and biomarker data will be sufficient to determine the feasibility of implementing the package of interventions and tracking youth sexual behaviors prospectively.

### Study Population

The eligibility criteria for MP3 Youth are shown in Textbox 2.

**General eligibility requirements for the pilot study.**

Criteria

- Inclusion criteria
  - Any male or female between the ages of 15 to 24 years.
  - Able to understand spoken English, Kiswahili, or Dholuo.
  - Willing to give informed consent (including emancipated minors), or, if younger than 18 years of age, has a parent or guardian willing to provide consent in addition to the minor’s assent.
  - Willing to be tested for HIV.
  - Willing to get a participant identification (ID) based on biometric finger scanning.
- Exclusion criteria
  - Any male or female younger than 15 years or older than 24 years of age.
  - Unable to understand spoken English, Kiswahili, or Dholuo.
  - If under 18 years of age, not an emancipated minor, and unable to get parental consent.

### Target Sample Size

As stated previously, the MP3 Youth package will be offered to approximately 1000 youth who attend the mobile events. These participants will be part of the cross-sectional analyses. In addition, a subset of youth will be enrolled in longitudinal cohorts. The cohorts and their corresponding sample sizes are shown in Textbox 3.

**Cohorts and sample sizes.**

Cohorts

- HIV-positive cohort:
  - All participants who test HIV positive (including participants who report being positive at baseline and are retested) during all mobile events will be invited to enroll in the longitudinal cohort using SMS/USSD surveys to collect data.
  - Participants will be given mobile phones.
  - The target HIV-positive sample size is approximately 100.
- Conditional cash transfer (CCT) cohort:
  - In-school girls between 15 to 24 years of age who test HIV negative will be screened for eligibility at each of the mobile events.
  - The target CCT sample size is approximately 50.
- Pre-exposure prophylaxis (PrEP) cohort:
  - Out-of-school girls who test HIV negative and are between 18 and 24 years of age will be screened for eligibility at each of the mobile events.
  - Participants will be given mobile phones.
  - The target PrEP sample size is approximately 50.
- HIV-negative cohort:
  - A random selection of participants who test HIV negative at the mobile events will be asked to complete one follow-up phone call at 12 months to assess willingness and intention to undergo HIV retesting (no study phone will be provided).
  - The target sample size is approximately 100.

### Study Outcomes

Study outcomes were developed by an expert panel during the formative phases of protocol development to evaluate the objectives of the study and the aforementioned methods employed to deliver the interventions (see Multimedia Appendix 1). The primary and secondary outcomes of MP3 Youth are shown in Textbox 4.

**Primary and secondary outcomes of the MP3 Youth protocol.**

Outcomes

- Primary study outcomes (cross-sectional and cohort)
  - Intervention uptake (acceptability) and coverage (feasibility)
  - Coverage
    - The proportion of youth in the community who attend each mobile event (estimated from youth population denominator)
  - Enrollment
    - The number of participants who consent to being enrolled in the study during each mobile event.
  - Uptake
    - The proportion of eligible participants who choose one or more components of their tailored combination package (including combinations of interventions components)
  - Intervention acceptability
    - Satisfaction with mobile event services (computer-assisted personal interview at exit from mobile event)
- Secondary study outcomes
  - Adherence to medication (HIV-positive and pre-exposure prophylaxis cohort participants only)
    - Adherence to once daily Truvada (pre-exposure prophylaxis, PrEP) among HIV-uninfected eligible females and patterns of adherence and sexual HIV acquisition risk exposure (PrEP cohort participants only).
    - Measured by self-report, electronic medication vial, and clinical assessments: monthly for the first 6 months and every 3 months thereafter and dried blood spot for analysis of tenofovir disoproxil fumarate/emtricitabine and tenofovir diphosphate and emtricitabine triphosphate at month 2 and 9.
  - Adherence to antiretroviral therapy (ART) (HIV-positive cohort participants only)
    - Measured by quarter annual text messages/unstructured supplementary service data self-report at 0, 3, 6, 9, and 12 months.
    - Point of care cluster of differentiation 4 (CD4) and baseline viral load by dried blood spot or plasma will be measured at mobile event baseline and repeated at 12 months.
  - Feasibility of administering conditional cash transfer to keep girls in school over 12 months.
    - Measured by school attendance (self-report and random checks for school attendance verification).

### Data Collection

This section of the protocol outlines the components that need to be included in order to collect and evaluate the cross-sectional and longitudinal data required to measure the pilot study outcomes. All MP3 Youth mobile event participants who are eligible, consent, and register for MP3 Youth will be required to complete a baseline behavioral CAPI survey and an exit acceptability interview. Participants who are eligible and enrolled in a sub-cohort will be required to complete SMS/USSD mobile phone surveys every month (PrEP cohort) or every 3 months (CCT and HIV-positive cohort). Participants in the PrEP cohort will be required to complete monthly clinical/laboratory visits for the first 6 months and every 3 months thereafter. The following sections describe the specific types of data that will be collected and the suggested data platforms to be implemented.

#### Behavioral Baseline Interview

The behavioral survey will be a staff-administered CAPI at the mobile events. For sensitive questions (which will be identified in the survey), youth will be instructed that they can tap on the answer with the tablet facing away from the interviewer and the answer will not be visible after entry; this incorporates elements of “ballot box response” or *audio computer-assisted self-interview* (ACASI) approaches while reducing issues around lack of literacy and unfamiliarity with tablet computers [62].

The CAPI will collect the following types of information, including but not limited to the types of services the participant is most interested in accessing: (1) basic demographic information, (2) sexual risk behaviors, (3) HIV risk behaviors, (4) attitudes and/or knowledge about HIV transmission and prevalence, (5) knowledge and acceptability of specific HIV prevention interventions, (6) knowledge and acceptability of family planning methods, and (7) attitudes towards the delivery of services (collected at the exit interview).

The CAPI will provide data that will lead to a better understanding of the combination HIV prevention interventions and prevention behaviors of youth. The responses will help determine the package of services for which the participant is eligible.

#### Follow-Up Text Message and Unstructured Supplementary Service Data Survey

Using the EchoMobile platform, study staff will make SMS/USSD text contact using an agreed-upon code, to ensure the phone user is the study participant. Staff will then wirelessly transfer airtime, so the cohort participant can send surveys using SMS/USSD [63]. The SMS/USSD survey will be a brief 10-question survey tailored to the cohort population. The surveys will be short to reduce barriers to completion. The responses will be analyzed to assess change in behavior over time as a part of the longitudinal cohort.

The SMS survey will collect information that includes but is not limited to (1) sexual risk behaviors; (2) school attendance (for CCT cohort); (3) adherence barriers and facilitators (PrEP and HIV-positive cohort); and (4) status disclosure (HIV-positive cohort).

#### Biometric Data Collection

Biometrics will be used to correctly identify participants, track repeat visits, and protect subject privacy and information. There will be a tablet connected via *Universal Serial Bus* (USB) to a biometric (fingerprint) scanner at the mobile events and during follow-up visits. This technology will be used to track uptake of services and to exclude individuals who try to enroll multiple times. The fingerprint software will translate a fingerprint into a code containing numbers and letters; no image of the fingerprint will be stored. In testing this procedure, we will take several measurements of correctly positioned clean fingers to ensure accurate measurement and re-measurement. The code will not be a personal identifier and it will not be possible to use it to recreate a fingerprint. These codes will be stored separately from other data. This technology has been used successfully in other projects in Kenya [64].

#### Biomedical Data Collection

Urine specimen will be collected for pregnancy testing for women with reproductive capacity and for dipstick analysis of protein and glucose levels (PrEP cohort only). In the PrEP cohort only, blood specimens will be collected for (1) HIV rapid testing, with confirmatory testing if one or both tests are reactive; (2) complete blood count (CBC); (3) serum creatinine (for estimated creatinine clearance); (4) serum phosphate; (5) aspartate transaminase (AST)/alanine transaminase (AST) ratios; (6) hepatitis B surface antigen (HepBsAg) (ineligible for PrEP if positive); (7) hepatitis B surfance antibody (HepBsAb) and hepatitis B Core antibody (HepBCore Ab) testing (if HepBsAg is negative); and (8) DBS for study drug levels as a measure of adherence. Blood specimens will also be collected to determine the CD4 cell count in any participant with confirmed HIV infection and viral load testing.

#### Implication of Pilot Study Results

Data from Aim 3 (the pilot study), the mathematical modeling sensitivity analyses, and the population impact projections will help establish the acceptability, feasibility, potential synergies or antagonisms, safety, and potential efficacy of the package before moving to a larger-scale evaluation phase IV study design (Aim 4).

### Aim 4: Combination Prevention Effectiveness Study Design

Using lessons from Aims 1 to 3 of the protocol, we present a preliminary outline for the design of a future effectiveness trial of the MP3 Youth package. The objective of the effectiveness trial will be similar to the pilot, but the trial will focus on effectiveness and not on feasibility.

Arguably, insufficient attention has been given to moving phase III efficacy trial interventions to phase IV effectiveness designs applied in heterogeneous real-world settings [65]. Several study designs can be considered to evaluate actual population-level impact of gender-specific combination prevention intervention packages for youth (Table 1), including cluster randomized controlled trials (cRCTs) using parallel or stepped wedge (ie, staggered) assignment. The advantage of cRCTs over individually randomized controlled trials (iRCTs) is that the former allows evaluation of the total effect of a combination package, especially if the sum of the intervention package components is greater than its parts [66]. They would also allow assessment of targeted interventions to subpopulations (eg, in the case of MP3 Youth males vs females or pregnant vs non-pregnant females) while the impact would be measured on the whole community. The feasibility of tracking an HIV incidence outcome for either an iRCT or cRCT would depend on development of improved cross-sectional HIV incidence assays. It would also depend on the expected HIV incidence in the control arm and likely HIV-incidence reduction effect size in the combination arm.

**Table 5:** Potential designs for a testable combination HIV prevention intervention study protocol.

Study design	Advantages	Disadvantages	Considerations
iRCT^a^	Rigorous control of confounders	Can’t evaluate population HIV impact	Need high incidence; control condition can weaken intervention detection
cRCT^b^	Rigorous, can assess impact beyond individual	Cost, complexity	Unit of randomization, and number of clusters and individuals within clusters
cRCT stepped wedge randomization	Easier to add interventions, once shown effective	Implementation delays can reduce effectiveness and power	Appropriate when logistically difficult to roll-out prevention service all at once
Program demonstration	Could be done in large scale in existing programs	Less rigorous, results can be inconclusive	Selection/matching of intervention and control communities; incidence measures

^a^iRCT: individually randomized controlled trial.

^b^cRCT: cluster randomized controlled trial.

We can use findings from Aims 1 to 3 to inform choice of (1) study design (including standard of care elements); (2) high HIV incidence setting (in Kenya or elsewhere) with sufficient HIV events expected; and (3) HIV incidence measurement for a full-scale evaluation of a gender-specific youth combination prevention package. Other key considerations that can be reviewed include what the trial unit of randomization should be, standard of care and/or comparator, timeframe expected for seeing an intervention effect, length of follow-up period needed to observe sufficient number of new HIV infections, sample size, participant inclusion/exclusion criteria, and how best to engage active and ongoing community involvement in large-scale trial design and conduct. Safety considerations and adverse event tracking must be delineated. We must also consider broader issues of the logistics of program coverage, cost-effectiveness, and sustainability beyond the study period. After compiling the possible options, we will collaborate with our research team to review our recommended study elements and make final trial design recommendations.

### Human Participants

The MP3 Youth study has institutional review board (IRB) approval from Kenyatta National Hospital/University of Nairobi-Ethics and Research Committee (KNH-ERC) and from New York University’s governing IRB, University Committee on Activities Involving Human Subject (UCAIHS).

## Results

Quantitative data analysis will be conducted using Stata software [67] and R statistical software [68]. Initial analyses will involve inspecting frequency distributions to identify outlying values and skewed variables requiring transformation. Descriptive analyses will provide means, medians, or prevalence of risk factors with associated confidence intervals. Analytic comparisons of pilot cohort drop-outs versus retained subjects will be done using multivariate analysis of variance (MANOVA) for normally distributed continuous variables with log10 (X+1) transformations on skewed behavioral variables, and chi-square tests for categorical variables and Wilcoxon tests for ranked data, with significance levels adjusted for multiple testing.

For the PrEP, CCT, and HIV-positive cohorts, changes over time in risk behaviors will be estimated focusing on initial change (baseline vs 6 months) and delayed change or persistence of initial effects (6 months vs 12 months). We anticipate minimal missing data due to use of CAPI and SMS/USSD data. The primary goal of the longitudinal cohorts is to provide data needed to describe the feasibility of PrEP, ART, and CCT, including medication adherence and school attendance over time. Levels of and changes in adherence and school attendance will be presented graphically, for example, with box plots at each assessment point and trajectory plots. For a detailed analysis plan, see Multimedia Appendix 2.

## Discussion

### Principal Findings

The MP3 Youth study designed and created a protocol to pilot and evaluate a gender- and youth-specific combination HIV prevention study in Kenya. We propose that offering youth a combination of HIV prevention interventions in a mobile health setting will be convenient and effective based on our preliminary research. Our formative research indicated that youth are interested in receiving multiple services in one place. We anticipate that the results of the exit interview we will conduct at the mobile events will yield positive feedback on the delivery of the interventions as the events will be conveniently located and youth friendly. Cross-sectional analyses of the mobile health data will provide insights into the uptake of interventions and feasibility of offering them in a one-stop-shop integrated services delivery modality. The results of the longitudinal cohort data will provide innovative information on the potential impact of the interventions and the behavioral changes among the cohort participants over time. The pilot study will give new insights into how components do or do not fit together in a real-world combination prevention package. The results of the pilot will then be used to refine the design of an effectiveness trial or a large-scale program evaluation.

MP3 Youth is one of the few combination HIV prevention interventions incorporating elements of behavioral, biomedical, and structural interventions in one delivery setting and developed for youth. Despite the fact that youth are both at highest risk for HIV and the largest proportion of the global population, only recently have they been targeted for comprehensive HIV prevention interventions.

The mobile health event strategy outlined in this protocol to identify youth is unique. We were able to use the information gleaned from focus groups to target the location and delivery of services and to use the literature and lessons learned from previous studies to design a protocol to deliver evidence-based interventions that are youth-friendly and gender-sensitive. The logistics of implementing mobile health events for 10 days at a time will involve setting up and breaking down a clinic that is completely equipped to handle HIV testing, CD4 and viral load analysis, family planning, a surgical theatre for male circumcision, and a pharmacy. This strategy will be effective for reaching youth who are at risk and in need of a variety of services. We will enroll participants using biometrics. This strategy will allow us to track participant flow and uptake of services during the mobile events. Biometrics will also allow us to successfully identify participants during follow-up visits [69].

### Limitations

This is a pilot feasibility study using mixed-methods (mathematical modeling, qualitative data, cross-sectional, and longitudinal cohort elements). As this pilot study does not employ a controlled trial design or comparator, population-level effectiveness of the combination HIV prevention approach cannot be determined. The logistics required to implement a combination HIV prevention study may be expensive and may require a lot of up front staff and referral partner capacity building that may delay implementation.

### Conclusion

The MP3 Youth lessons learned will provide real-world contributions to implementation science regarding uptake of combination prevention. Some of the preliminary lessons learned in developing the protocol were considered as part of the Kenya national health strategy; one of the 8 countries participating in the PEPFAR DREAMS initiative. The analyses conducted for MP3 Youth will be key in highlighting evidence for a scaled-up youth prioritized HIV/AIDS strategy in Kenya and other high HIV burden settings.

## Appendix

Multimedia Appendix 1 Logic Model.

Multimedia Appendix 2 Evaluation plan.

## Abbreviations

ART: antiretroviral therapy
CAPI: computer-assisted personal interview
CCT: conditional cash transfer
CD4: cluster of differentiation 4
cRCT: clustered randomized controlled trial
DBS: dried blood spots
eCAP: electronic medication vial
HepBsAg: hepatitis B surface antigen
HIV: human immunodeficiency virus
IRB: institutional review board
iRCT: individually randomized controlled trials
PrEP: pre-exposure prophylaxis
SSA: sub-Saharan Africa
TDF/FTC: tenofovir disoproxil fumarate/emtricitabine
USSD: unstructured supplementary service data
