# Supplementary figures and images for: Gender-Specific Combination HIV Prevention for Youth in High-Burden Settings: The MP3 Youth Observational Pilot Study Protocol
Source: JMIR Res Protoc. 2017 Mar 8;6(3):e22. doi: 10.2196/resprot.5833 (PMC5434770; doi:10.2196/resprot.5833)

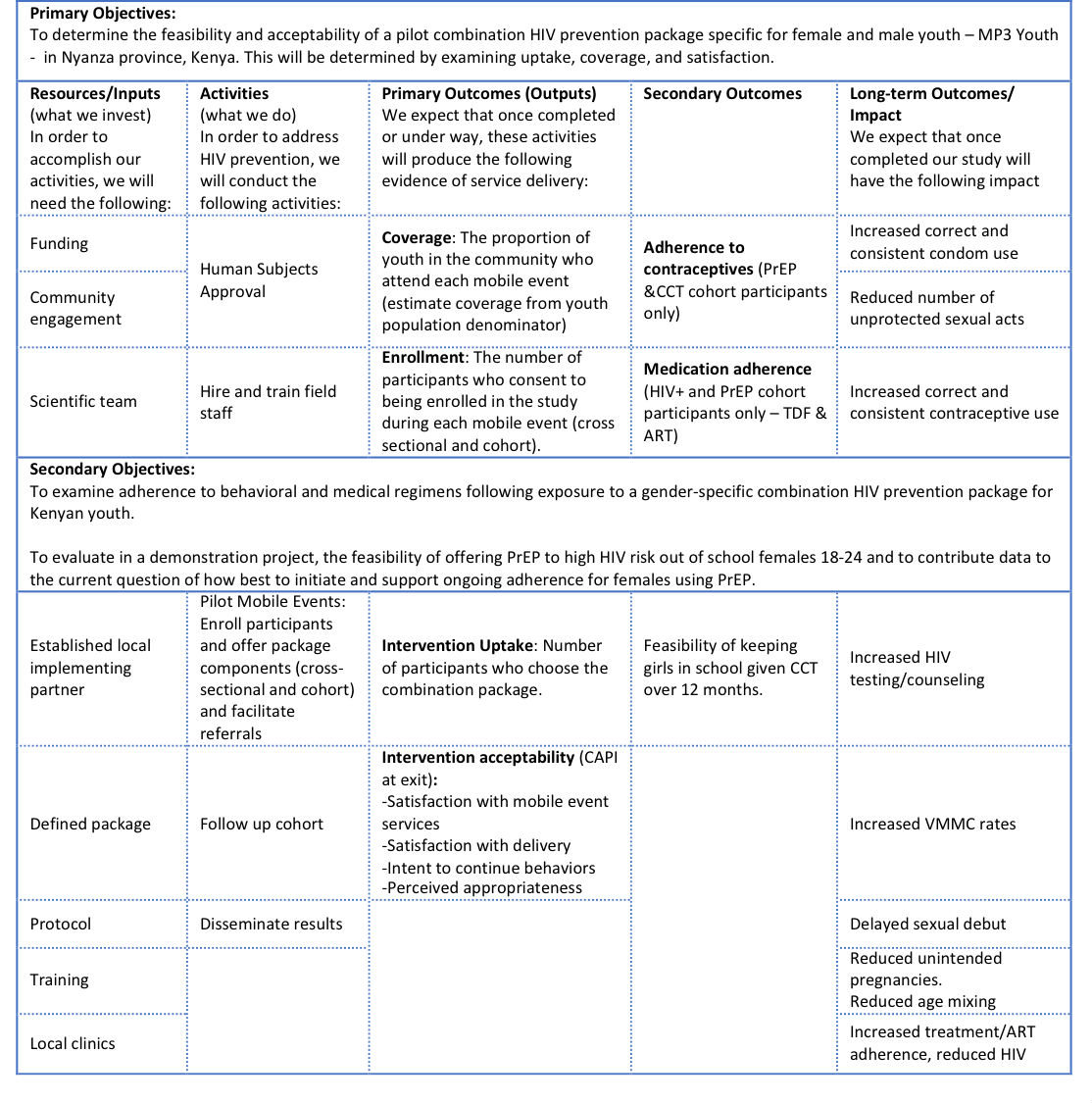

Supplement: Multimedia Appendix 1 [file resprot_v6i3e22_app1.jpg]

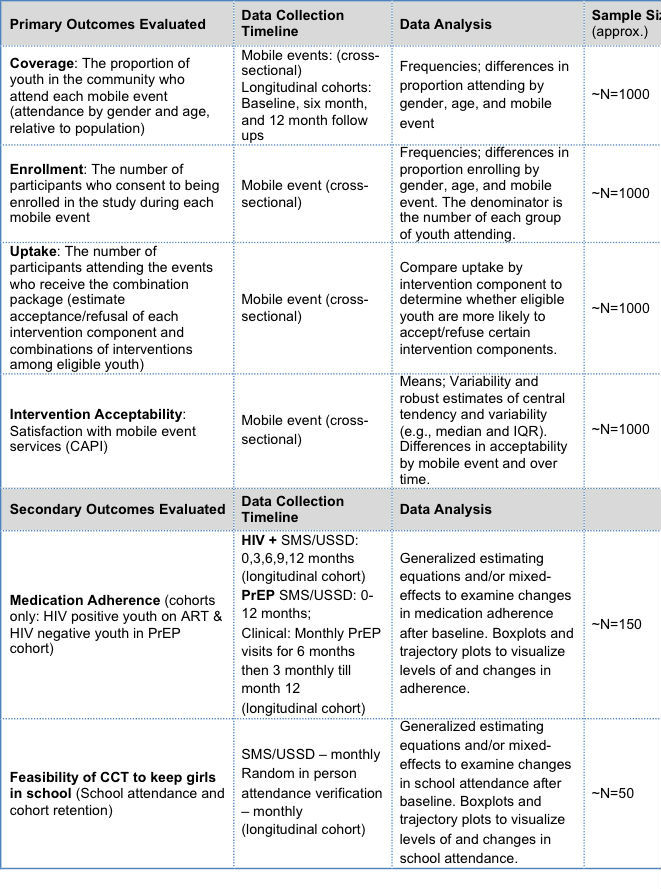

Supplement: Multimedia Appendix 2 [file resprot_v6i3e22_app2.jpg]
